# Paneth cells as orchestrators of epithelial barrier defense and emerging therapeutic targets in inflammatory bowel disease

**DOI:** 10.1007/s00281-026-01068-x

**Published:** 2026-03-16

**Authors:** Lena Erkert, Lea-Maxie Haag, Christoph Becker

**Affiliations:** 1 Department of Medicine 1, Universitätsklinikum Erlangen, Friedrich-Alexander-Universität Erlangen-Nürnberg, Erlangen, Germany; 2 Department of Gastroenterology, Infectious Diseases and Rheumatology (including Nutrition Medicine), Charité – Universitätsmedizin Berlin, corporate member of Freie Universität Berlin and Humboldt-Universität zu Berlin, Berlin, Germany; 3 Deutsches Zentrum Immuntherapie (DZI), Erlangen, Germany

**Keywords:** Paneth cells, Intestinal epithelial barrier, Gut microbiome, Inflammatory bowel disease

## Abstract

First described by Joseph Paneth in 1888 in the small intestine, particularly in the crypts of Lieberkühn, Paneth cells have since emerged as a critical subtype of intestinal epithelial cells (IECs), which together constitute the body’s largest interface with the external environment, continuously exposed to pathogens, dietary components, and toxins. Paneth cells represent a unique, long-lived secretory IEC population located at the crypt base, where they play indispensable roles in antimicrobial defense and stem cell niche maintenance. Their differentiation, positioning, and survival are governed by tightly regulated signaling networks, including the Wnt and Notch pathway. Although traditionally viewed as terminally differentiated, emerging evidence suggests Paneth cells possess a certain level of plasticity, enabling functional adaptation or dedifferentiation under stress or injury. These characteristics position Paneth cells as central regulators of intestinal homeostasis and epithelial barrier integrity. Over the last decades, accumulating evidence has established that Paneth cell dysfunction is closely linked to microbial dysbiosis and the development of inflammatory bowel disease (IBD), highlighting their contribution to disease pathogenesis. Recent discoveries on how Paneth cell dysfunction contributes to intestinal inflammation are uncovering new therapeutic approaches aimed at reestablishing Paneth cell homeostasis and alleviating IBD progression. In this review, we comprehensively summarize current knowledge on Paneth cell differentiation, function, and their role in gut host defense and epithelial barrier maintenance. We further discuss mechanisms by which Paneth cell dysfunction disrupts intestinal homeostasis, promoting IBD development, and highlight emerging therapeutic strategies that target Paneth cells to reestablish barrier integrity and restore gut health.

## Epithelial barrier and microbiota

### Structure and function of the epithelial barrier

**Fig. 1:**
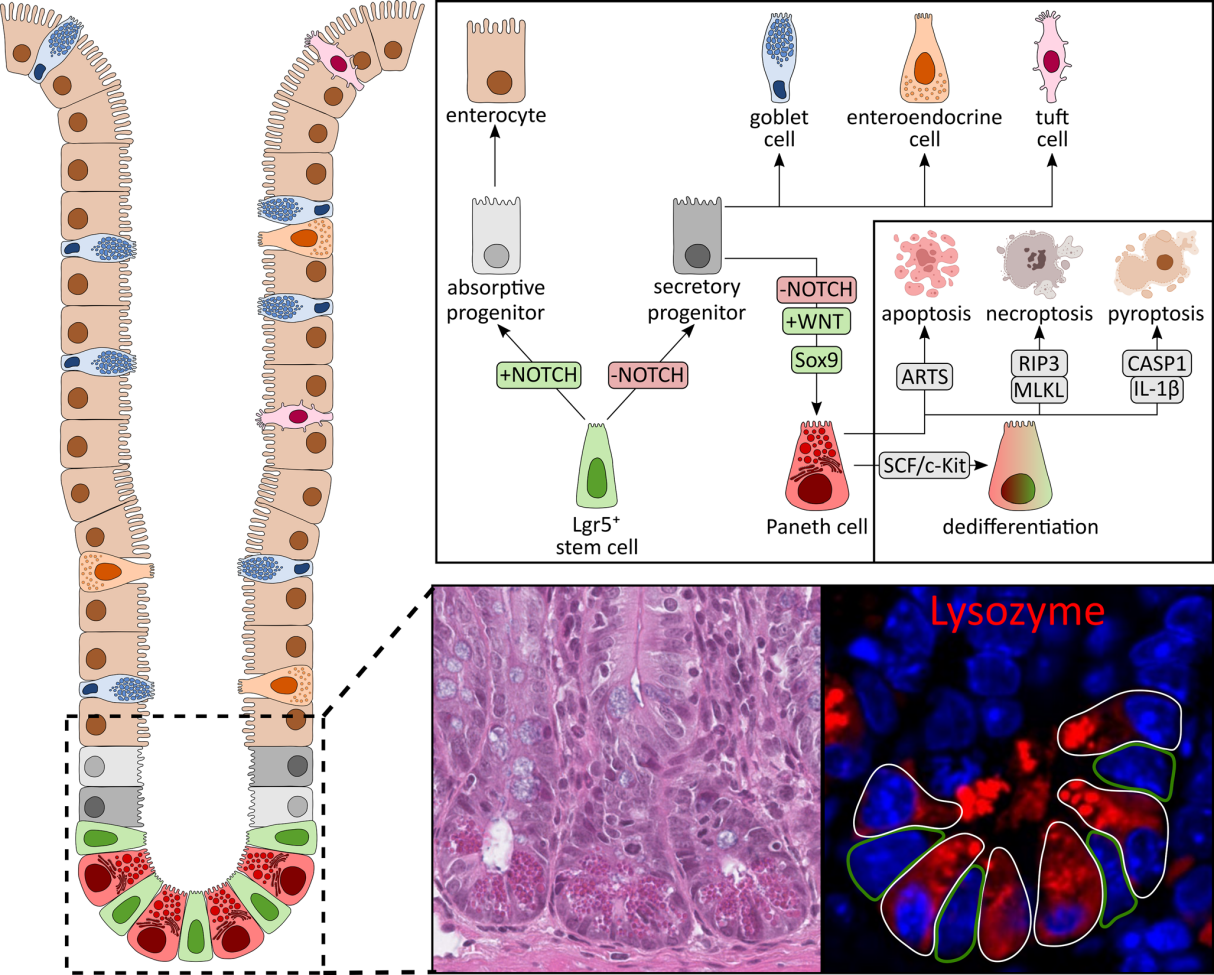
Paneth cell differentiation. Lgr5^+^ stem cells located at the crypt base generate rapidly proliferating progenitor cells in the lower crypt, which subsequently differentiate into mature epithelial lineages. The early differentiation of Lgr5^+^ stem cells is primarily regulated by Notch signaling. Cells receiving Notch activation develop into absorptive enterocytes, whereas those lacking Notch activity adopt a secretory progenitor fate. Among secretory progenitors, cells that maintain Wnt signaling migrate back to the crypt base and mature into Paneth cells, while those that do not receive Wnt signals differentiate into goblet, enteroendocrine, or tuft cells. Within the crypt base, mature Paneth cells are interspersed among Lgr5^+^ stem cells in a nearly geometric pattern that optimizes Paneth cell-stem cell interactions. Mature Paneth cells are identifiable by lysozyme expression, a hallmark antimicrobial peptide they produce and secrete. Following their lifespan, Paneth cells may undergo various forms of cell death – apoptosis under homeostatic conditions, or necroptosis and pyroptosis during intestinal stress and inflammation. Alternatively, they can dedifferentiate into stem-like cells, contributing to epithelial regeneration

The intestinal epithelial barrier provides a tight defense against luminal contents. This is achieved by continuous renewal of a single epithelial cell layer every three to five days in the small intestine, ensuring optimal function and preventing pathology caused by damaged cells [1]. Beyond barrier function, intestinal epithelial cells (IECs) act as a communication hub between the gut microbiota and immune cells in the lamina propria [2]. At the crypt base, Lgr5^+^ intestinal stem cells (ISCs) give rise to all specialized IEC types, which can be divided into two groups, absorptive enterocytes and secretory cells including goblet, Paneth, enteroendocrine, and tuft cells [3, 4] (Fig. 1). ISCs first generate transit-amplifying cells, which undergo several divisions in the upper crypt before differentiating into mature IECs and migrating toward the surface. Upon reaching the surface, cells detach and undergo cell death [4]. Neighboring IECs rapidly reseal gaps by reforming tight junctions within 20 min, preventing pathogen intrusion [5]. This coordinated turnover, termed physiological cell shedding, maintains both epithelial numbers and barrier integrity [1]. IECs are primarily interconnected by adherens junctions and tight junctions, which together form the apical junction complex [6]. Adherens junctions, mainly composed of E-cadherin and catenins, mediate the initial cell-cell adhesion and are a prerequisite for the subsequent assembly of tight junctions. In contrast, tight junctions are formed by claudins, occludin, and junctional adhesion molecules that are linked to scaffolding proteins like ZO-1. These structures seal the paracellular space and act as signaling hubs, regulating permeability, polarity, and immune responses [7]. At the molecular level, multiple intestinal signaling pathways coordinate a tightly regulated yet flexible process that drives IEC differentiation while preserving barrier integrity and preventing disease. For example, Notch signaling is central for cell fate decisions, where secretory progenitors activate Notch in neighboring progenitors, promoting their differentiation into absorptive enterocytes [8]. Notch also sustains stemness, with Paneth cells providing the major ligands Delta-like protein 1 (Dll1) and Dll4 [9], while ISCs express the Notch receptors Notch1 and Notch2 [10]. In contrast, Wnt/β-catenin signaling, particularly Wnt3 secreted by Paneth cells, is essential for ISC proliferation [11]. Similarly, Paneth cell-derived epidermal growth factor (EGF) drives ISC expansion via EGFR [12]. Conversely, BMP and Hedgehog signaling peak at the villus tips and restrain crypt proliferation, ensuring proper differentiation [13, 14].

**Absorptive enterocytes**, comprising more than 80% of IECs, form a brush border specialized for nutrient absorption [15]. **Goblet cells**, accounting for 10–15% of IECs in the small intestine and up to 50% in the colon [16], secrete mucus that acts as a first-line barrier and facilitates luminal transit [15]. In the small intestine, a single, loosely attached mucus layer is present, whereas the colon contains two distinct mucus layers, an outer layer that is permeable to bacteria and an inner, tightly adherent layer that is impermeable to bacteria [17]. Mucus thickness increases distally along the gut [18] and its gel-like properties arise from highly O-glycosylated mucins [19]. Among mucins, MUC2 is the principal gel-forming mucin in both the small intestine and colon. In contrast, transmembrane mucins form a carbohydrate-rich surface layer known as the glycocalyx and include MUC3, MUC13, and MUC17 in the small intestine, and MUC1, MUC3, MUC6, MUC12, MUC13 and MUC17 in the colon [20]. Stored in secretory granules, mucins are continuously released or their secretion can be stimulated by factors such as neuropeptides, cytokines, or lipids [21]. **Paneth cells** as another secretory IEC type and the central focus of this review, will be discussed in detail later. Other secretory IECs include **enteroendocrine cells**, which comprise approximately 1% of IECs and are dispersed along the gut [15]. Different enteroendocrine cell subtypes produce specific hormones and peptides regulating secretion, motility, food intake, glucose homeostasis, and metabolism [22]. **Tuft cells**, though rare and less well understood, act as chemosensory sentinels that detect luminal signals and contribute to immune defense, notably via secretion of IL-25 and eicosanoids [23]. In contrast, microfold **(M) cells** neither absorb nor secrete molecules. Instead, they transport luminal antigens to antigen-presenting cells such as dendritic cells in underlying lymphoid follicles, initiating mucosal immune responses [24].

Overall, proper ISC differentiation into diverse IEC types, coupled with balanced proliferation and shedding, is essential to preserve barrier integrity and intestinal homeostasis.

### Microbiota

The intestinal epithelium, with its specialized cell types, is crucial for preventing access of commensal and pathogenic microorganisms from the lumen. The microbiota, encompassing 10–100 trillion microbes in the gut, includes bacteria, fungi, archaea and viruses that interact with the host through symbiosis, commensalism, pathogenicity, or as pathobionts [25]. Pathobionts are commensal microorganisms that normally coexist with the host without causing harm but can become pathogenic and contribute to disease under specific genetic or environmental perturbations [26]. The composition of the microbiota varies along the gastrointestinal tract, with microbial load and diversity increasing from the proximal small intestine to the distal colon [27]. While the duodenum harbors ~ 10³ bacteria per gram of intestinal contents, the ileum contains ~ 10⁷, and the colon up to ~ 10¹² per gram [28]. The microbiota is highly dynamic, differing between individuals and fluctuating within the same host [29]. Microbial diversity typically increases from infancy to adulthood and may decline in older age, although this varies with health status and lifestyle [30]. Genetics contribute, but environmental factors, including diet, medications, hygiene, and infection, play a dominant role in shaping microbiota composition [30]. Most members of the microbiota are commensal and not overtly pathogenic. Although the functions of many individual species remain poorly characterized [31], the microbiota as a whole contributes to host health by (1) aiding nutrition, (2) limiting pathogen growth, and (3) modulating and maturing immune cells.

In the context of **nutrition**, the microbiota ferments non-digestible dietary components to generate short-chain fatty acids (SCFAs), such as butyrate, acetate, and propionate, along with amino acids and vitamins. These metabolites are essential for host nutrition and drive intestinal epithelial growth and differentiation [32]. Germ-free mice highlight this role, as they display reduced intestinal surface area, shorter ileal villi, and smaller crypts due to diminished epithelial turnover, compared to conventionalized mice [33]. Microbiota-mediated regulation of epithelial renewal involves microbial-associated molecular patterns, such as lipopolysaccharide (LPS), which signal through Toll-like and Nod-like receptors in IECs [34].

Pathogen control is achieved via “**colonization resistance**”, whereby commensals outcompete pathogens for nutrients, occupy attachment sites, and secrete antimicrobial compounds including bacteriocins and SCFAs [35]. They also modulate luminal pH and oxygen, creating hostile conditions for pathogens [36]. Importantly, resistance depends more on microbiota composition than total microbial abundance [37].

The microbiota also shapes **immune system development** through interactions with gut-associated lymphoid tissue, such as Peyer’s patches, which contain IgA-producing plasma cells, and T lymphocytes. Macpherson et al. showed that CD11c^+^ dendritic cells in Peyer’s patches sample bacterial antigens, inducing secretory IgA-producing B cells that restrict commensal growth and penetration [38]. Intestinal epithelial lymphocytes, positioned between epithelial cells, are crucial for immune tolerance toward symbionts [39]. Germ-free or antibiotic-treated mice further underscore this dependency. They exhibit underdeveloped mucosal and lymphoid structures, such as fewer Peyer’s patches, smaller mesenteric nodes, absence of germinal centers, and reduced isolated follicles, with diminished antibody production and diversity [40].

As discussed below, antimicrobial peptides secreted by Paneth cells also shape microbial composition, thereby influencing barrier integrity and host health.

## Paneth cells: key players in intestinal barrier regulation

### Paneth cell differentiation

Paneth cells are specialized IECs found in many species, including mice, rats, chickens, non-human primates, and humans. However, they are either absent or morphologically indistinct in species such as pigs, sheep, and cows, where other cell types may perform similar antimicrobial functions [41]. In mice and rats, Paneth cells appear postnatally, around days 7–10, coinciding with crypt formation [42], whereas in humans they are detectable much earlier, at approximately 13.5 weeks of gestation [43]. Paneth cells are restricted to the small intestine and, under homeostatic conditions, absent from the colon. Their abundance increases progressively from the proximal to the distal small intestine by three- to seven-fold, likely reflecting the greater need for bactericidal activity in the distal small intestine [44]. For example, in the jejunum, Paneth cells comprise approximately 1% of all IECs [45]. Mature Paneth cells are defined by expression of lysozyme, matrix metalloproteinase 7 (MMP7), and CD24 [46].

Paneth cells are the only secretory IEC subtype that **remains at the crypt base** rather than migrating along the crypt-villus axis. They reside among Lgr5⁺ ISCs, where their distribution follows an almost geometric arrangement that maximizes heterotypic (Paneth-ISC) contacts while minimizing homotypic (Paneth-Paneth) contacts [12] (Fig. 1). This positioning is enabled by their lack of ephrin-B ligands, which normally drive upward migration of other IEC types through interaction with EphB receptors in the proliferative zone [47]. Unlike other differentiated IECs, which are short-lived (3–5 days), Paneth cells have a lifespan of nearly two months [48]. Paneth cells exhibit a pyramidal morphology with basally located nuclei and an extensive endoplasmic reticulum (ER) and Golgi network, reflecting their high secretory output [49]. Their apical surface contains large secretory granules, which deliver antimicrobial peptides, lysozyme, defensins, and cytokines in response to both microbial stimuli such as LPS [50], as well as to microbe-independent stimuli, including host cell-derived factors such as IFN-γ [51]. Notably, the smallest Paneth cells, with small granules, are located adjacent to the ISC zone, whereas larger Paneth cells with abundant granules are positioned at the crypt base, intermingled with ISCs [52]. The earliest sign of differentiation is the appearance of lysozyme within the Golgi complex, followed by progressive accumulation in secretory granules. As Paneth cells age, these granules gradually diffuse into the cytoplasm [53].

**Differentiation of ISCs into Paneth cells** depends on a precise interplay between genetic programs and signaling pathways (Fig. 1). Wnt/β-catenin signaling is essential for both Paneth cell specification and their downward migration to the crypt base. The transcription factor TCF4, a major effector of Wnt/β-catenin signaling, is required for Paneth cell maturation during mouse embryogenesis [54]. In this context, *Tcf4*-deficient mice exhibit defective crypt development and a complete absence of mature Paneth cells [55]. Disruption of Wnt targets further illustrates this requirement. For example, deletion of EphB3, a Wnt-responsive receptor, impairs spatial cues and results in Paneth cell scattering along the crypt-villus axis [56]. Similarly, loss of Frizzled-5 or APC impairs Paneth cell differentiation, leading to mislocalization or ectopic Paneth cell formation in the colon [54, 57]. Transcription factors also regulate Paneth cell fate. Sox9 is indispensable, as its deletion eliminates Paneth cells without affecting other IEC lineages [58]. *Spdef* deficiency results in accumulation of immature progenitors, blocking maturation of both Paneth and goblet cells [59]. Importantly, Wnt signaling must be active while Notch signaling must be repressed for Paneth cell commitment. Genetic models disrupting Notch signaling (e.g., IEC-specific deletion of *Rbpj* [60], *Adam10* [61], or *Hes1/Hes3/Hes5* [62]) consistently increase Paneth cell numbers. In line with this, simultaneous deletion of presenilins (Psen1/2), key components of the γ-secretase required for Notch receptor cleavage, induces massive Paneth cell hyperplasia and mislocalization along the crypt-villus axis [63]. Moreover, environmental factors can also modulate Paneth cell differentiation. For example, infection with *Trichinella spiralis* promotes new Paneth cell production by displacing older cells toward the crypt base border [64].

Although long-lived, Paneth cells ultimately undergo programmed **cell death** (Fig. 1). Under homeostatic conditions, they primarily die by apoptosis, an immunologically silent process facilitated by the apoptosis-related protein in TGF-β signaling (ARTS). Loss of ARTS prolongs Paneth cell survival, leading to increased numbers [65]. In contrast, during intestinal inflammation, several studies have described necroptosis (a pro-inflammatory form of cell death) in Paneth cells [66, 67]. Paneth cells express receptor-interacting protein kinase 3 (RIP3), sensitizing them to necroptosis [66], a process strongly implicated in inflammatory bowel disease (IBD). In addition, Paneth cells can undergo pyroptosis, a caspase-1-dependent inflammatory cell death pathway. Following γ-irradiation, Paneth cells activate caspase-1 and release IL-1β, triggering pyroptosis [68]. Beyond cell death, Paneth cells exhibit remarkable plasticity. Under certain conditions, they can **dedifferentiate into multipotent stem cells** (Fig. 1). In dextran sodium sulfate (DSS)-induced colitis, Paneth cell dedifferentiation is driven by SCF/cKit signaling via PI3K/Akt and Wnt pathways [69]. While Notch inhibition is required for Paneth cell differentiation, Notch activation can conversely trigger dedifferentiation. In a doxorubicin-induced injury model, Notch signaling enabled *Defa4*-expressing Paneth cells to dedifferentiate into ISC-like cells, thereby promoting intestinal regeneration [70]. Similar observations were made following irradiation, where activation of Notch signaling endowed Paneth cells with stem cell-like properties [71]. Collectively, these findings underscore the remarkable plasticity of Paneth cells, whose signaling and functional adaptability are crucial for maintaining intestinal barrier integrity.

### Paneth cell function

**Fig. 2:**
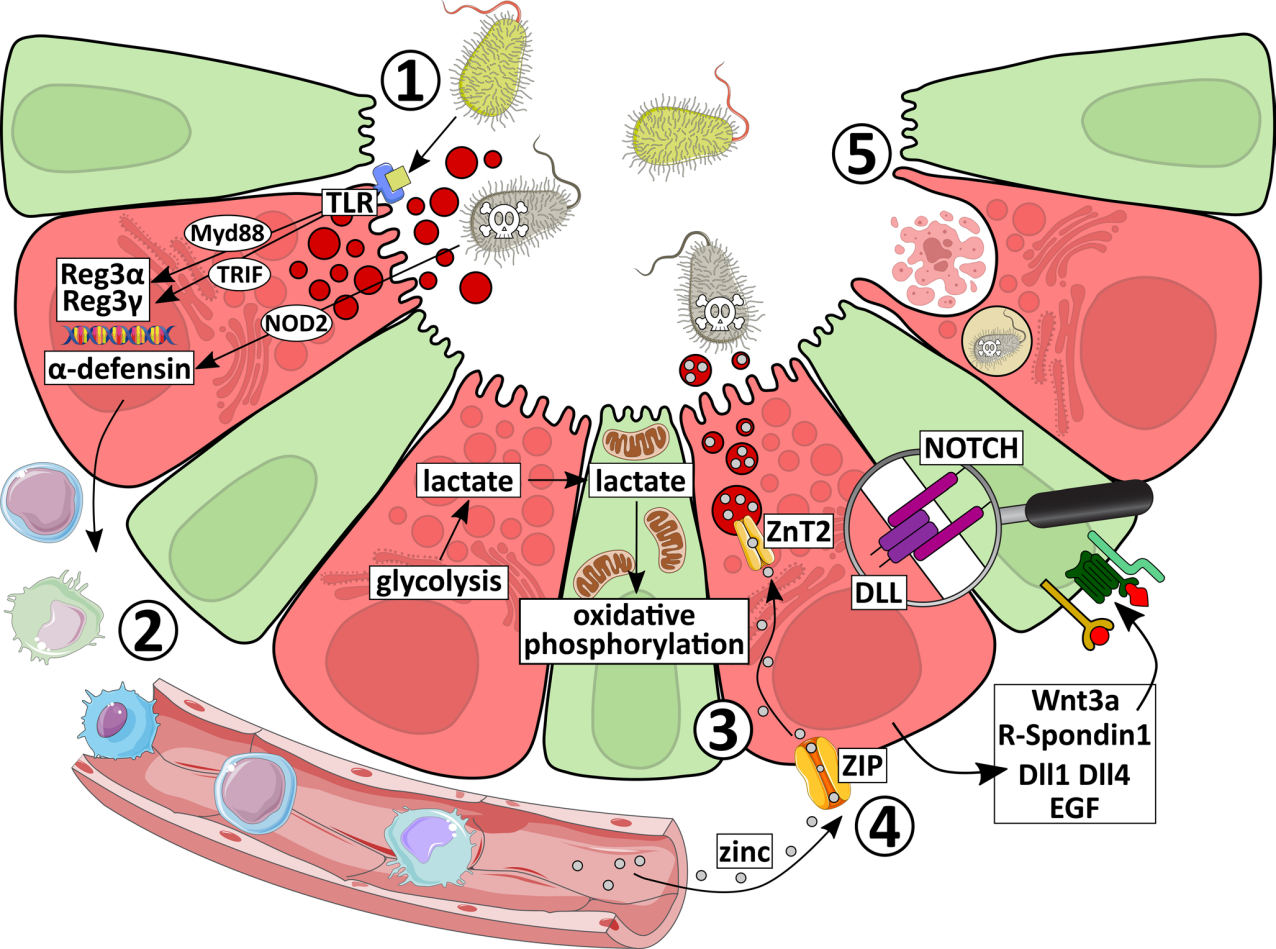
Paneth cell functions under homeostatic conditions. Paneth cells perform multiple essential functions to preserve intestinal epithelial integrity and host defense under homeostatic conditions: (1) Paneth cells detect bacterial components through intracellular NOD2 and surface-expressed Toll-like receptors (TLRs), activating downstream signaling via MyD88 or TRIF. These pathways induce the secretion of antimicrobial peptides (AMPs), which exert diverse bactericidal mechanisms. (2) In addition to their antimicrobial activity, several AMPs act as chemoattractants, recruiting immune cells to sites of microbial challenge. (3) Located at the crypt base and intermingled with intestinal stem cells, Paneth cells provide essential niche factors that promote stem cell maintenance, including Wnt ligands (e.g., Wnt3a), R-spondin 1, Notch ligands (Dll1, Dll4), and epidermal growth factor (EGF). Moreover, Paneth cells support stem cell function by supplying lactate that fuels enhanced mitochondrial oxidative phosphorylation in stem cells, leading to mitochondrial reactive oxygen species-dependent signaling and thereby promoting the mature crypt phenotype. (4) Uptake of essential trace metals, such as zinc, by Paneth cells are important for their secretory function and contribute directly to antimicrobial defense. (5) Finally, Paneth cells help stabilize the crypt microenvironment by clearing apoptotic cells through efferocytosis and by directly phagocytosing invading bacteria

The most extensively characterized function of Paneth cells is the regulation of gut microbiota composition through secretion of antimicrobial peptides and proteins. Beyond this, as detailed in the following chapter, Paneth cells also provide essential support to ISCs, carry out phagocytosis and efferocytosis, participate in metal uptake, and exert immunomodulatory functions. Together, these functions are essential for preserving epithelial barrier integrity and overall gut homeostasis (Fig. 2).

#### Secretory functions of Paneth cells

As mentioned before, the main function of Paneth cells is the continuous production and secretion of antimicrobial peptides (AMPs), positioning them as key regulators of gut microbiota composition and mucosal defense. Through the secretion of these small peptides (~ 4 kDa), Paneth cells contribute vitally to intestinal barrier function and homeostasis by preventing both commensal and pathogenic bacteria from crossing the epithelial barrier.

Paneth cells produce a unique repertoire of **AMPs**, including enteric α-defensins (called cryptidins in mice), lysozyme, regenerating islet-derived protein 3 (REG3), secretory phospholipase A2 (sPLA2), and Angiogenin-4 (ANG4) (Table 1). These AMPs are stored in secretory granules and released at the apical side of the Paneth cell into the crypt lumen [72] (Fig. 2). Notably, Paneth cells in mice contain six cryptidin isoforms, while human Paneth cells express only two (HD5 and HD6) [44]. This difference complicates the use of conventional knockout models to study α-defensin function in mice. Moreover, genetic variability in α-defensin loci across mouse strains must be taken into account when investigating Paneth cell biology in mice [73]. Another species difference is that, while human neutrophils can also produce α-defensins (HNP1-4) [74], mouse neutrophils do not [75]. Interestingly, IgA has been detected within Paneth cell granules, although it is not synthesized by Paneth cells but rather derived from IgA-secreting plasma cells in the lamina propria and secondarily taken up into Paneth cell granules [76]. Secreted α-defensins and other AMPs are concentrated in the mucus layer to strengthen the mucosal barrier [77]. Thereby, the highest concentrations of estimated 15–100 mg/ml are found in close proximity to the ISCs, optimally protecting them from microbial intrusion [50]. Lysozyme, the first AMP discovered in Paneth cells and still a widely used Paneth cell marker, is expressed as a single isoform (Lysozyme C) in humans but as two isoforms in mice, Lysozyme C type P (*Lyz1*; secreted by Paneth cells) and type M (*Lyz2*; secreted by macrophages) [78]. Similar to lysozyme, sPLA2 is primarily constitutively expressed and is also secreted by macrophages [79]. By contrast, REG3 (REG3α in humans and REG3γ in mice, as the predominant isoforms expressed in Paneth cells [80]) and ANG4 are inducibly expressed [81]. Among all AMPs, α-defensins are the most abundant, accounting for about 70% of Paneth cell-derived AMPs and being largely constitutively expressed [50].

**Table 1:** Antimicrobials produced by Paneth cells

Antimicrobial	Present in human/mouse	Bactericidal property against…	Bactericidal function	Constitutive/induced expression
α-defensins	Mouse: 6 cryptidins Human: 2 (HD5, HD6)	Mainly gram-positive and -negative bacteria	Perforation of microbial membrane	Constitutive
Lysozyme	Mouse: Lysozyme C type P (Paneth cells; *Lyz1*), type M (macrophages; *Lyz2*) Human: Lysozyme C	Gram-positive bacteria	peptidoglycan hydrolyzation	Constitutive
sPLA2	Mouse: *Pla2g2a* (absent in C57BL/6) Human: *PLA2G2A*	Mainly gram-positive bacteria	phospholipid hydrolyzation	Constitutive
C-type lectins	Mouse: REG3γ Human: REG3α	Gram-positive bacteria	Binds bacterial peptidoglycans ◊ forms membrane-permeabilizing pores	Induced
ANG4	Mouse: ANG4 Human: not present	Gram-positive and -negative bacteria	Disruption of bacterial membrane integrity	Induced

*HD* human defensin, *sPLA2* secretory phospholipase A2, *REG3* regenerating islet-derived protein 3, *ANG4* Angiogenin 4

The **production of α-defensins** occurs in a two-step process to limit cytotoxicity to host cells. First, pre-pro-peptides are synthesized and transported from the ER into secretory vesicles, where the signal peptide is removed to generate pro-peptides. These are subsequently processed into active peptides by proteolytic cleavage [82]. In humans, this maturation is carried out by luminal trypsin [83], while in mice it is mediated by MMP7, which is co-secreted with the pro-α-defensins [84]. Accordingly, *Mmp7*^*−/−*^ mice lack mature cryptidins and instead accumulate precursors, resulting in an altered intestinal microbiota, increased bacterial loads during *Salmonella typhimurium* infection, and more severe inflammatory symptoms [84]. In contrast, overexpression of HD5 in mice confers protection against *S. typhimurium* [85], underscoring the importance of α-defensins for barrier function and host health.

The **degranulation** of AMP-containing granules from Paneth cells is regulated by a variety of microbial and host-derived factors. Paneth cell secretion is triggered by a range of stimuli, including live Gram-positive and Gram-negative bacteria, bacterial-associated molecular patterns such as LPS and muramyl dipeptide (MDP; a peptidoglycan component), and cholinergic agonists like carbamylcholine, which mimic neuroimmune signaling. In contrast, fungal and protozoal components generally do not induce degranulation [50]. In addition, cytokines including IL-4, IL-13 [86], IL-17 A, IL-22 [87], IFN-α [88], IFN-γ [51], and TNF-α [89] have all been shown to induce degranulation, as have microbial-derived SCFAs [90]. Several **modes of secretion** have been described. Under homeostatic conditions, Paneth cells probably rely predominantly on merocrine secretion, in which granules are released by exocytosis [41]. In contrast, during inflammatory conditions such as IFN-γ stimulation, Paneth cells undergo holocrine secretion, resulting in the extrusion of the entire Paneth cells into the lumen [51]. In addition, lysozyme can be secreted via secretory autophagy, an alternative pathway activated by bacteria-induced ER stress [91]. Remarkably, AMP release is extremely rapid, occurring within 2–14 s after apical stimulation with LPS in enteroid models. Granule contents are replenished within approximately 21 h, allowing Paneth cells to maintain continuous surveillance and defense of the stem cell niche [92].

AMPs act mainly through **bacterial membrane disruption** (Table 1). For example, α-defensins, enriched in basic amino acids such as histidine, lysine, and arginine, carry a positive charge that enables electrostatic interaction with anionic bacterial membrane components [93]. Lysozyme hydrolyzes peptidoglycan in bacterial walls [94], sPLA2 preferentially targets phosphatidylethanolamine and phosphatidylglycerol [95], abundant components of bacterial membranes, while ANG4 exerts bactericidal activity by disrupting membrane integrity by interacting with bacterial membranes via its basic amino acid residues [96]. α-defensins and ANG4 act against Gram-negative and Gram-positive bacteria, whereas lysozyme, sPLA2, and C-type lectins are mainly active against Gram-positive species [97]. Notably, α-defensins are the only Paneth cell-derived AMP with confirmed antiviral activity [98]. Importantly, animal studies further show that AMPs preferentially target non-commensal bacteria, thereby sparing the beneficial microbiota [99].

Recent work identified two Paneth cell subtypes distinguished by the expression of fucosyltransferase 2 (Fut2). Fut2⁺ Paneth cells, predominantly located in the ileum, display higher granularity and structural complexity and constitute the main source of AMPs in the gut, while Fut2⁻ Paneth cells are enriched in the duodenum [87]. Interestingly, despite being secreted in the small intestine, mature defensins remain stable and functionally active throughout the gastrointestinal tract, including the colon [100].

Finally, it is important to note that enteric pathogens have developed **strategies to evade Paneth cell-derived AMPs**. These strategies include reducing the net negative charge of their cell envelope (primarily through covalent modifications of LPS and other anionic lipid-sugars [101]), expelling AMPs via energy-dependent efflux pumps, altering membrane fluidity, or degrading AMPs with proteases. Moreover, a common strategy employed by many bacteria is the production of surface-associated polysaccharide capsules that enhance in vivo survival by sequestering cationic AMPs and limiting their access to the bacterial cell surface [102]. Such mechanisms illustrate the evolutionary pressure exerted by Paneth cell defenses and the capacity of pathogens to adapt [103].

#### Mechanisms of Paneth cell response to the microbiota

Paneth cells sense bacteria and initiate downstream signaling primarily through cell-autonomous activation of toll-like receptors (TLRs) in a myeloid differentiation primary response 88 (MyD88)-dependent manner, as well as through nucleotide-binding oligomerization domain-containing protein 2 (NOD2) (Fig. 2). Both pathways induce the expression of AMPs, although they display distinct preferences for specific subsets [104, 105]. For example, expression of REG3α and REG3γ is dependent on the **TLR-MyD88-axis**, as Myd88^−/−^ mice lack REG3γ expression [101], likely due to impaired IL-22 induction. Remarkably, by Paneth cell-specific reconstitution of MyD88 (via Defa2-MyD88 transgenic expression) AMP production was restored and the phenotype was reversed [104]. Functionally, when infected with *S. typhimurium*, Defa2-MyD88 transgenic mice harbored fewer bacteria in their mesenteric lymph nodes than Myd88^−/−^ control mice [106], demonstrating that Paneth cell-intrinsic MyD88 signaling is sufficient to restore intestinal barrier defense. Paneth cell degranulation can be induced by multiple TLR agonists, including polyinosinic: polycytidylic acid (TLR3), LPS (TLR4), Flagellin (TLR5), and CpG-oligodeoxynucleotides (TLR9). The kinetics of these responses differ – TLR3 and TLR9 ligands trigger a rapid degranulation, whereas TLR4 and TLR5 stimulation leads to delayed responses mediated by TNF-α [89]. While TLR4, TLR5, and TLR9 signal via MyD88, TLR3 requires the adaptor molecule Toll-IL-1 receptor domain-containing adaptor molecule 1 (TRIF) [107]. Unlike C-type lectins, which predominantly signal through MyD88, α-defensins are strongly dependent on **NOD2** (a MDP receptor), as evidenced by reduced expression of multiple α-defensins in NOD2^−/−^ mice [108, 109]. In addition to regulating α-defensins, NOD2 signaling is essential for proper lysozyme handling. In NOD2^−/−^ mice, lysozyme undergoes abnormal degradation, indicating that NOD2 contributes to its stabilization or proper granule trafficking [110]. These alterations in NOD2^−/−^ mice ultimately result in significant changes in the small intestinal microbiota composition [111]. Remarkably, overexpression of HD5 in NOD2^−/−^ can effectively restore crypt antimicrobial activity and ultimately rescues the inflammatory phenotype following *Helicobacter hepaticus* infection [112].

Supporting this link between AMPs and microbial ecology, fecal α-defensin-5 concentrations positively correlated with beneficial taxa such as *Ruminococcaceae*, *Allobaculum*, *Sutterella*, and *Akkermansia*, while negatively correlating with potentially harmful taxa including *Erysipelotrichaceae* [113]. Two different **mouse models** have been particularly informative for dissecting AMP effects on microbiota composition – *Mmp7*^*−/−*^ mice, which lack mature cryptidins [84], and *DEFA5* (HD5) transgenic mice, which express HD5 specifically in Paneth cells under the *DEFA5* promoter [85]. Notably, while overall bacterial load remains unchanged, *Mmp7*^*−/−*^ mice exhibit increased *Firmicutes* and decreased *Bacteriodetes*, whereas *DEFA5* transgenic mice show the opposite effects, with decreased *Firmicutes* and increased *Bacteriodetes* [106]. However, it is important to note that MMP7 is expressed in multiple cell types and tissues, and therefore the observed microbiota and Paneth cell-related phenotypes in Mmp7^−/−^ mice warrant cautious interpretation.

Collectively, these findings demonstrate that Paneth cells, through MyD88- and NOD2-mediated sensing pathways and subsequent AMP release, play a central role in shaping the baseline intestinal microbiota. In turn, this microbial equilibrium sets the stage for both mucosal and systemic immune responses.

#### Non-secretory functions of Paneth cells

Beyond their well-characterized secretory role, Paneth cells also engage in a variety of non-secretory functions that critically impact intestinal immunity and homeostasis.

The anatomical location of Paneth cells at the crypt base, intermingled with ISCs, reflects their intimate functional relationship and their essential role in **supporting the intestinal stem cell niche**. Within this niche, Paneth cells secrete a range of factors that are indispensable for ISC maintenance, including Wnt ligands, R-Spondin 1, EGF, Notch ligands, and transforming growth factor α (TGF-α). Together, these factors sustain ISC self-renewal, promote proliferation, and maintain the undifferentiated state of stem cells [12]. In this context, a central role is played by Wnt signaling. Paneth cells provide multiple Wnt ligands (Wnt3a, Wnt9b, Wnt11) that activate Frizzle receptors on ISCs, driving Wnt/β-catenin signaling [11, 12, 114, 115]. R-Spondin 1, secreted by Paneth cells, further amplifies Wnt signaling by binding LGR4/5 on ISCs [116]. Notably, the ability of Paneth cells to support ISCs diminishes with organismal aging, as aged Paneth cells secrete less Wnt3a and instead release the Wnt inhibitor Notum, thereby impairing ISC renewal [117]. Besides Wnt-related signals, Paneth cells contribute to ISC proliferation through the secretion of EGF, and regulate stem cell numbers via Notch signaling. Thereby, Paneth cell-expressed membrane bound ligands Dll1 and Dll4 interact with Notch receptors (mainly Notch1 and Notch2) on ISCs to sustain their expansion [12]. Metabolic interactions between Paneth cells and ISCs also play a crucial role. Paneth cells supply lactate, which sustains the enhanced mitochondrial oxidative phosphorylation in ISCs (Fig. 2). Consequently, inhibition of glycolysis in Paneth cells disrupts ISC function [118]. Interestingly, Paneth cell support of ISCs is further influenced by the microbiota. For instance, *Bifidobacterium* spp. and *Lactobacillus* spp. produce lactic acid, which stimulates Paneth cells through the G-protein-coupled receptor Gpr81 to upregulate Wnt3a, thereby enhancing Wnt/β-catenin signaling in ISCs [119]. Remarkably, in the absence of Paneth cells, other secretory epithelial lineages such as enteroendocrine and tuft cells can partially compensate by providing the essential niche factors [120]. As explained above, Paneth cells exhibit remarkable plasticity, as they can redifferentiate through SCF/c-Kit signaling and contribute directly to the ISC pool, thereby promoting epithelial regeneration [69].

Paneth cell-derived AMPs not only exert direct antimicrobial activity but also act as **chemoattractants for immune cells** (Fig. 2). For instance, human α-defensin 5 induces migration of macrophages, mast cells, and both naïve and memory T cells, but not immature dendritic cells [121]. While the receptors mediating α-defensin-induced chemoattraction on immune cells remain unidentified, they appear to be distinct from those used by β-defensins [121]. The latter act through chemokine receptors such as CCR2, which mediates macrophage recruitment [122], and CCR6, involved in the attraction of immature dendritic cells and memory T cells [123]. The importance of Paneth cell-derived AMPs in immune activation is highlighted in *Mmp7*^*−/−*^ mice, which lack functional α-defensin maturation and thus fail to develop germinal centers during enteric infection [124]. In addition, Paneth cells themselves produce cytokines. For example, TNF-α has been detected to accumulate in their secretory granules [125], and IL-17 – previously thought to be exclusively immune cell-derived – is also stored in Paneth cell granules [126]. Notably, TNF-α stimulation enhances IL-17 secretion from Paneth cells, driving TNF-α-induced small intestinal injury and Paneth cell dysfunction, characterized by secretory granule loss, mitochondrial dilation, and autophagic vacuole accumulation [126, 127]. Conversely, TNF-α release from Paneth cells can be triggered by endotoxin exposure, though the physiological role of Paneth cell-derived TNF-α in intestinal homeostasis remains to be clarified [128]. However, it could be hypothesized that Paneth-cell derived TNF-α may act as a rapid, epithelial-derived alarm signal during microbial invasion, complementing their antimicrobial peptide secretion and activating nearby immune cells in the lamina propria to amplify early innate immune responses.

Beyond secretion, Paneth cells also contribute to microbial clearance through cell-intrinsic mechanisms, including phagocytosis (Fig. 2). Electron microscopy studies have identified spiral-shaped bacteria within Paneth cell digestive vacuoles, some of which appeared partially digested, providing direct evidence of phagocytic uptake [129]. In addition, Paneth cells can perform **efferocytosis**, an immunologically silent form of phagocytosis that clears apoptotic cells and is proposed to occur via autophagy, thereby contributing to an anti-inflammatory intestinal environment [130] (Fig. 2).

Another important, but not exclusive, non-secretory function of Paneth cells lies in the **uptake of essential trace metals**, such as zinc (Fig. 2). Zinc uptake is indispensable for Paneth cell function. Accordingly, loss of the zinc importer ZIP4 leads to abnormal Paneth cell gene expression and a disruption of intestinal integrity [131], while deletion of the zinc exporter ZnT2 (exports the absorbed zinc towards the secretory granules) in mice disrupts granule formation, reduces antimicrobial activity, and promotes dysbiosis, all mediated through Paneth cell dysfunction [132]. Zinc is thought to support Paneth cell activity by stabilizing lysozyme [132], exerting direct antimicrobial toxicity [133], and enabling MMP7 activity during the final proteolytic maturation of α-defensins [134]. Importantly, zinc is also required for Paneth cell survival, as chelation of zinc causes rapid Paneth cell loss [135].

## Pathological conditions: Paneth cell dysfunction and barrier breakdown in Crohn’s disease

As described above, under physiological conditions, Paneth cells reside exclusively in the small intestine. However, in IBD, Paneth cell-like metaplastic cells can also appear in the colon, likely reflecting an adaptive epithelial response to strengthen antimicrobial defense at comprised sites [136].

IBD encompasses two main entities, ulcerative colitis (UC) and Crohn’s disease (CD). While UC is confined to the colon – a region normally devoid of Paneth cells – CD typically involves both ileum and colon. IBD is a multifactorial, chronic inflammatory disorder driven by genetic susceptibility, dysregulated immune responses to intestinal microbiota, and environmental influences that alter microbial composition. Importantly, a primary disruption of the epithelial barrier can also permit bacterial invasion, triggering uncontrolled immune activation and chronic inflammation [137].

Genome-wide association studies have identified multiple Paneth cell-related genes that increase CD susceptibility (Table 2). Interestingly, these mutations predominantly reduce AMP expression rather than impairing secretion. Of note, Paneth cell abnormalities are found in 20–50% of CD patients with a higher prevalence in pediatric CD patients than in adult patients [138].

**Table 2:** IBD-Paneth cell-associated susceptibility genes

GENE	PATHWAY AFFECTED	PHENOTYPE IN KNOCKOUT MICE	CONSEQUENCES IN IBD PATIENTS	REFERENCES
NOD2	Nod-like receptor signaling	• Decreased α-defensin levels • Lysozyme degradation • Microbial dysbiosis • CD-like granulomas upon *H. hepaticus* infection	• Decreased α-defensin levels	[109]
ATG16L1	autophagy	• Abnormalities in granule exocytosis pathway • High levels of ER stress • Accumulation of dysfunctional mitochondria • Increased susceptibility to bacteria-induced inflammation	• Diminished PC granules • SNP: ATG16L1^T300A^	[142, 143, 145, 146]
ATG5				
IRGM		• Abnormal PC sizes • Dysregulation of PC localization • Less dense granules • Increased susceptibility to DSS colitis	• Only association described	[148, 150, 151]
LRRK2		• Impairment of lysozyme expression in PC	• Less PC numbers • Diminished, diffuse and excluded PC subtypes • SNPs: G2019S, N2081D	[110, 155]
XBP1	Unfolded protein response	• PC loss • Spontaneous enteritis development (ER stress) • Susceptible to DSS-induced colitis	• Only association described	[156, 158]
CASP8	necroptosis	• Necroptosis of PCs	• Necroptosis of PCs	[66]
TCF1	Wnt signaling	• Reduced α-defensin expression	• Reduced α-defensin expression	[160]
TCF4		• Reduced α-defensin expression • Decrease in bacterial killing activity		[161]
LRP6		• No PC phenotype		[162]

*CD* Crohn’s disease, *ER* endoplasmatic reticulum, *PC* Paneth cell, *SNP* single nucleotide polymorphism, *DSS* dextran sodium sulfate

***NOD2***, one of the earliest CD susceptibility genes identified, is highly expressed in Paneth cells where it regulates α-defensin (HD5, HD6) expression [109], and AMP sorting [139]. Interestingly, in the absence of NOD2, SPF mice remain healthy under normal conditions. However, when infected with *Helicobacter hepaticus*, NOD2-deficient mice develop characteristic CD-like granulomatous lesions in the distal small intestine [112]. This phenotype results from a defect in the C-terminal leucine-rich repeat domain of NOD2, which normally recognizes MDP and mediates bacterial sensing in the ileum. Loss of this function promotes aberrant microbial colonization and subsequent intestinal inflammation [140]. Consistently, CD patients carrying *NOD2* mutations exhibit markedly reduced ileal α-defensin expression [141].

Multiple studies have shown that defects in the **autophagy** pathway – a lysosomal degradation system for damaged organelles, proteins and pathogens – impair Paneth cell number and function, reflecting their dependence on autophagy for secretion regulation. In this context, deletion or mutation of autophagy-related genes such as autophagy related 16 like 1 gene (***ATG16L1***) or ***ATG5***, which together with ATG12 form the ATG12-ATG5-ATG16L1 complex, causes marked abnormalities in granule exocytosis, diffuse lysozyme staining [142], elevated ER stress [143], and accumulation of dysfunctional mitochondria in Paneth cells [142]. While *Atg16l1* knockout mice are neonatally lethal [144], mice carrying an *Atg16l1* T300A knock-in variant (resulting in functional destabilization of ATG16L1) display increased susceptibility to bacteria-induced inflammation [145], mirroring Paneth cell granule defects observed in CD patients carrying the ATG16L1^T300A^ risk variant [146]. Beyond ATG16L1, mitochondrial dysfunction in general has been linked to gut microbiota dysbiosis and early Paneth cell abnormalities, ultimately resulting in the spontaneous development of ileitis [147]. Similarly, loss of the autophagy-related protein ***IRGM*** results in abnormal Paneth cell sizes, mislocalized Paneth cells with less dense granules, and increased susceptibility to DSS-induced colitis [148]. Compared with NOD2 and ATG16L1, the contribution of IRGM to IBD pathogenesis is less clearly defined and appears more context dependent. Genetic studies first identified common *IRGM* variants as CD susceptibility loci [149, 150], which are associated with altered IRGM expression rather than coding changes [151]. IRGM regulates selective autophagy by interacting with the core autophagy machinery [152] and modulating mitochondrial dynamics [153], thereby limiting proinflammatory signaling such as NLRP3 inflammasome activation [154]. Through these effects on epithelial stress responses, microbial handling, and Paneth cell homeostasis, IRGM is thought to contribute to intestinal inflammation without a single dominant mechanistic pathway. In addition, Leucine-rich repeat kinase 2 (***LRRK2***) deficiency disrupts lysozyme expression in Paneth cells through aberrant autophagic degradation [110]. Consistently, Crohn’s disease patients carrying LRRK2 variants show a reduced number of Paneth cells per crypt and display granules that are either absent, morphologically abnormal, or lack stainable contents [155].

Similar to autophagy, the **unfolded protein response** (UPR), a cellular pathway that resolves ER stress, is critical for Paneth cell maturation and function. For example, Kaser et al. demonstrated that mice lacking the transcription factor X-box-binding protein 1 (**XBP1**) exhibit reduced lysozyme and α-defensin staining, indicative of Paneth cell loss due to ER stress [156]. XBP1 maintains ER function and expansion, which is essential for the proper functioning of highly secretory cells such as Paneth cells [157]. Interestingly, IEC-specific deletion of *Xbp1* induces ER stress and apoptosis, leading to spontaneous small intestinal inflammation. This phenotype is characterized by heightened sensitivity to bacterial products (flagellin), inflammatory mediators (TNF-α), and Paneth cell dysfunction [156]. Dual deletion of *Atg16l1* and *Xbp1* in mice exacerbates this phenotype, resulting in severe CD-like transmural ileitis [158]. Importantly, several *XBP1* SNPs have been linked to CD pathogenesis in humans [156].

Remarkably, CD patients exhibit an increase in **necroptosis** of Paneth cells [66]. In mice, IEC-specific deletion of ***caspase-8*** leads to spontaneous Paneth cell necroptosis, which can be reversed by the necroptosis-inhibitor necrostatin-1 (Nec-1) [66]. Elevated levels of RIP3 – a key necroptosis mediator – have also been detected in Paneth cells from CD patients, implicating this pathway in ileal CD [66]. Notably, ATG16L1 prevents TNF-α-induced Paneth cell necroptosis by maintaining mitochondrial homeostasis – a process that can likewise be rescued by Nec-1 when ATG16L1 is absent [67]. Elevated IFN-λ levels in the serum and inflamed ileum of Crohn’s disease patients provide further evidence supporting necroptotic Paneth cell death. IFN-λ treatment activates STAT1 signaling and upregulates mixed lineage kinase domain-like protein (MLKL) – the terminal effector of necroptosis – thereby sensitizing Paneth cells to necroptosis, a process controlled by caspase-8 [159].

As discussed above, **Wnt signaling** is essential for the differentiation, maturation and positioning of Paneth cells. Accordingly, several Wnt-associated genes have been identified as susceptibility loci for CD. For example, mutations in ***TCF-1*** markedly reduced HD5 and HD6 expression, and overall TCF-1 levels are significantly decreased in CD patients [160]. Likewise, a ***TCF-4*** SNP has been linked to reduced α-defensin expression in CD patients [161]. Furthermore, variants in the Wnt co-receptor **LRP6** are associated with diminished HD5 production in Paneth cells and early-onset ileal CD [162].

Beyond genetic susceptibility, several **environmental factors** implicated in IBD pathogenesis also affect Paneth cell integrity. For example, high-fat [163] or Western-style diets [164], antibiotic exposure [165], or tobacco smoke [146] have all been shown to induce Paneth cell defects.

Remarkably, Paneth cell morphology has emerged as a **potential biomarker** and prognostic indicator for CD, aiding in disease classification and outcome prediction. Based on lysozyme-positive granule morphology, Paneth cells are categorized as normal, disordered, diminished, diffuse, or excluded [166]. CD patients with a higher proportion of dysmorphic Paneth cells exhibit significantly shorter relapse-free intervals after surgical resection compared to those with predominantly normal Paneth cells [166]. Moreover, an increased frequency of abnormal Paneth cells correlates with reduced bacterial diversity and a loss of anti-inflammatory microbial taxa [167].

While, as described above, Paneth cell defects are most prominently associated with CD, limited Paneth cell-related phenotypes have also been reported in UC patients. For example, Paneth cell metaplasia in the colon, a region normally devoid of Paneth cells, has been observed in 85% of pediatric UC patients [136], a finding further supported by data from an extensive integrated single-cell RNA sequencing database [168]. Moreover, an ileal abnormal Paneth cell phenotype has been suggested as a cellular biomarker for pouch complications in UC patients [169]. However, direct evidence that intrinsic Paneth cell defects contribute to UC pathogenesis is currently lacking.

Despite the well-documented associations in CD, it remains unclear whether Paneth cell dysfunction actively drives disease pathogenesis or arises secondary to environmental triggers – such as diet or antibiotic use – that induce microbial dysbiosis, promote intestinal inflammation, and exacerbate ER stress in Paneth cells, ultimately causing epithelial injury and Paneth cell loss [170].

Notably, in addition to Paneth cell metaplasia and dysfunction, Paneth cell hyperplasia has also been reported in CD patients, with increased Paneth cell numbers particularly observed in chronic active ileitis [171]. A similar Paneth cell hyperplasia phenotype has also been described in pediatric IBD patients [136]. Although this may appear counterintuitive at first glance, Paneth cell hyperplasia during chronic intestinal inflammation may reflect a regenerative response, as post-mitotic Paneth cells can acquire, as explained above, stem cell-like properties under inflammatory conditions and thereby contribute to epithelial repair [69].

## Therapeutic perspectives: targeting Paneth cells in IBD

Although Paneth cell dysfunction has been implicated in the pathogenesis of IBD, therapeutic strategies specifically targeting Paneth cells remain poorly established. Current treatment options for IBD primarily include long-established pharmacological therapies such as corticosteroids (e.g., prednisolone) and immunomodulators (e.g., methotrexate, azathioprine). In addition, a variety of biologic agents are used, including anti-TNF-α therapies (infliximab, adalimumab, certolizumab), anti-integrin agents (vedolizumab), and anti-IL-12/IL-23 monoclonal antibodies (ustekinumab). Moreover, emerging approaches such as probiotics, fecal microbiota transplantation, and dietary supplementation have shown promise as adjunctive strategies, particularly in modulating the gut microbiota and enhancing mucosal barrier function [172]. Despite these advances, therapeutic approaches directly targeting Paneth cell-driven pathogenic pathways remain limited. Paneth cell-directed therapeutic approaches would ideally aim to (1) preserve Paneth cell integrity and function or (2) enhance antimicrobial peptide secretion (Table 3).

**Table 3:** Potential Paneth cell-specific treatment options for IBD

TARGET	INTERVENTION	IMPACT IN IBD	MECHANISM	REFERENCE
Preservation of PC integrity and function	azathioprine	Upregulation of AMPs, enhances/restores PC differentiation and function	Restores mitochondrial function in PCs	[173]
	Mito-Tempo	Antagonizes PC dysfunction	Targets mitochondrial damage in PCs	[174]
	4-phenylbutyrate	Normalizes α-defensin levels	Inhibits ER stress	[175]
	TNF-α blocking antibody, Necrostatin-1s (RIPK1 inhibitor)	Antagonizes PC death	Inhibiting necroptosis in PCs	[67]
	Adalimumab (anti-TNF-α)			[176]
	Glucocorticoids, janus kinase inhibitors (tofacitinib, filgotinib)			[159]
	Nur77 agonists (BTP, Csn-B)			[177]
	Zinc			[178]
	Rosiglitazone (PPARγ activator)		Inhibiting apoptosis in PCs	[146]
	API5		API5 released from IELs promotes viability	[179]
	Dichloroacetate	Antagonizes PC dysfunction	Blocking glycolysis	[180]
	LRRK2 inhibitor		Activation of autophagy	[155]
Enhancement of AMP secretion	HD5 overexpression	Increased resistance to *S. typhimurium*	Antimicrobial defense	[85]
		Reversed inflammatory phenotype in *H. hepaticus*-infected NOD2^−/−^ mice		[112]
	Exogenous HD5	Improved dysbiosis, inflammation, and barrier defects		[182]
	HD5 produced by *Lactococcus lactis*	Reduced intestinal damage and inflammation in DSS-induced colitis		[183]
	AhR	Restores microbial gut balance, ameliorates colitis	Induction of α-defensin 1 expression	[185]
	CC34 (synthetic AMP)	downregulation of pro-inflammatory cytokines, reduced intestinal damage and inflammation	Inhibition of NF-κB	[186]
	DEFA1	Reduces severity of spontaneous enteritis and DSS-induced colitis	Antimicrobial defense	[187]
	Human β-defensin 2	Mitigates inflammation, improves disease activity index	Inhibition of NF-κB, increases CREB phosphorylation	[188]
	Brilacidin (phase 2 clinical trial)	Clinical remission induction in UC patients	Antimicrobial defense	[189]

*PC* Paneth cell, *AMP* antimicrobial peptide, *BTP* Bioactive triple peptide, *Csn*-*B* Cytosporone B, *API5* apoptosis inhibitor 5, *IELs* intestinal epithelial lymphocytes, *HD5* Human defensin-5, *AhR* aryl hydrocarbon receptor

A key goal is to restore Paneth cell homeostasis disrupted by mitochondrial dysfunction, ER stress or Paneth cell death, as described above. Several studies have already demonstrated that conventional IBD therapies exert part of their therapeutic effects by modulating Paneth cell function. For example, a recent study demonstrated that azathioprine promotes the upregulation of AMPs, enhances Paneth cell differentiation, and restores mitochondrial dysfunction-associated Paneth cell defects [173]. Consistent with that, as mitochondrial damage has been reported in Paneth cells of CD patients, treatment of CD biopsies with Mito-Tempo, a mitochondrial-targeted antioxidant, normalized pro-inflammatory cytokine expression (IL-17, IL-23), lipid metabolism, and apoptotic gene signatures to levels comparable with non-IBD controls [174]. ER stress similarly contributes to Paneth cell dysfunction. In this context, in obese individuals, bile acid-induced ER stress and defective autophagy impaired Paneth cell secretory granules and reduced α-defensin expression, a phenotype that was prevented by the ER stress inhibitor 4-phenylbutyrate [175]. Paneth cell necroptosis represents another key mechanism of epithelial injury. ATG16L1 deficiency, a major genetic risk factor for IBD, sensitizes Paneth cells to TNF-α-mediated necroptosis. Interestingly, therapeutic blockade of necroptosis through inhibition of TNF-α or RIPK1 signaling ameliorated intestinal inflammation in a virally triggered IBD model [67]. Supporting clinical relevance, IBD patients carrying the *ATG16L1 rs10210302* variant were more likely to require adalimumab therapy, as this polymorphism failed to prevent TNF-α-mediated Paneth cell necroptosis [176]. In related work, Cui et al. showed that Paneth cell-specific deletion of *Nur77* in mice exacerbated sepsis-induced intestinal inflammation due to impaired ER-phagy and increased Paneth cell necroptosis. Interestingly, treatment with Nur77 agonists such as bioactive triple peptide (BTP) or Cytosporone B (Csn-B) restored Paneth cell homeostasis [177]. Similarly, Zinc supplementation protected mice from TNF-α-induced Paneth cell necroptosis [178]. Environmental factors also influence Paneth cell integrity. Cigarette smoking induces Paneth cell defects in genetically susceptible hosts carrying ATG16L1^T300A^ alleles, which can be mitigated by pharmacological activation of PPARγ with rosiglitazone [146]. Additionally, therapeutic administration of recombinant apoptosis inhibitor 5 (API5) – normally secreted by γδ intestinal epithelial lymphocytes but impaired by *ATG16L1* mutation – protected Paneth cells from death in norovirus-infected *ATG16L1* knockout mice [179]. Additional studies suggest that suppression of IFNL-STAT1 signaling by glucocorticoids or pharmacological inhibition of Janus kinase signaling (e.g. filgotinib, tofacitinib) can block necroptosis and restore Paneth cell survival [159]. Furthermore, recent evidence indicates that targeting glycolytic metabolism with dichloroacetate can restore mitochondrial respiration, improve stem cell function, and antagonize inflammation-associated Paneth cell dysfunction [180]. Of note, due to toxicity concerns that limit the clinical applicability of dichloroacetate [181], the cited study should be regarded as a proof-of-concept demonstrating that targeting glycolytic metabolism may represent a potential strategy to antagonize Paneth cell dysfunction in CD. Finally, an indirect strategy to preserve Paneth cell homeostasis may involve the use of LRRK2 inhibitors, as LRRK2-driven pro-inflammatory cytokine release from phagocytes has been shown to impair Paneth cell function through autophagy activation [155].

Direct enhancement of AMP secretion represents a second therapeutic strategy to target Paneth cells in IBD pathogenesis. In this context, overexpression of HD5 in mice markedly increased resistance to *Salmonella typhimurium* infection [85]. Similarly, transgenic HD5 expression in Paneth cells reversed the Th1-driven granulomatous colitis phenotype observed in *NOD2* knockout mice infected with *Helicobacter hepaticus* [112]. Moreover, administration of exogenous HD5 also ameliorated colitis-associated dysbiosis, inflammatory responses and barrier defects in mice [182], while a continuous delivery of mature HD5 by genetically engineered *Lactococcus lactis* reduced intestinal damage and inflammation in DSS-induced colitis [183]. Clinically, HD5 upregulation has been observed in UC patients responding to anti-TNF-α therapy, supporting its contribution to treatment efficacy [184]. Recent findings further highlight transcriptional and synthetic strategies to boost AMP activity. For example, Palrasu et al. identified the aryl hydrocarbon receptor (AhR) as a transcriptional regulator of α-defensin 1. In a murine model of experimentally induced colitis, AhR-mediated induction of α-defensin 1 restored gut microbial balance and alleviated colitis symptoms [185]. In addition, the synthetic antimicrobial peptide CC34 demonstrated anti-inflammatory properties inhibiting NF-κB signaling and reducing the secretion of pro-inflammatory cytokines in an LPS-induced intestinal inflammation mouse model [186]. Likewise, administration of DEFA1 reduced spontaneous enteritis and DSS-induced colitis in *Atf4*-IEC knockout mice [187]. Moreover, human β-defensin 2 treatment in DSS colitis, as well as TNBS colitis mouse models was also able to mitigate intestinal inflammation by decreasing NF-κB signaling and increasing CREB phosphorylation [188]. Encouragingly, AMP supplementation is currently under clinical investigation. Brilacidin, a synthetic defensin mimetic derived from plant compounds, achieved clinical remission induction in UC patients and was generally well tolerated [189]. Despite all these advances, the clinical translation of AMP-based therapy remains limited. The main challenges of using AMP supplementation include ensuring AMP stability in the intestinal microenvironment, preventing protease-mediated inactivation, and improving delivery efficiency [190], all of which are subjects of ongoing investigation [191].

## Conclusion

Since their discovery more than 130 years ago, Paneth cells have emerged as remarkably multifunctional components of the intestinal epithelium. Traditionally recognized for their secretory function – particularly the release of antimicrobial peptides that directly shape the microbial community – Paneth cells are now known to exert a wide range of additional roles that extend far beyond antimicrobial defense. These include supporting neighboring intestinal stem cells through paracrine signaling, modulating immune responses via the chemoattractant properties of antimicrobials, and even performing phagocytosis and efferocytosis – functions that remain understudied but may hold therapeutic potential. Notably, Paneth cells also display a remarkable plasticity, with the ability to redifferentiate into intestinal stem cell-like cells under certain conditions. This capacity for epithelial regeneration represents an exciting avenue for therapeutic exploration, particularly in diseases characterized by epithelial damage, such as IBD. Recent technological advances – such as intestinal organoid systems, single-cell RNA sequencing, Paneth cell-specific sorting strategies and Cre-deleter mouse models – are now enabling more precise and comprehensive investigations of Paneth cell biology. These tools will be essential for unraveling the complexity of Paneth cell-microbiota interactions and their contribution to epithelial barrier homeostasis. Although numerous mouse studies have linked Paneth cell dysfunction to IBD pathogenesis and identified several Paneth cell susceptibility genes associated with CD, translation of these findings into Paneth cell-targeted therapies remains limited. Future research should prioritize bridging this gap by integrating experimental insights into clinical applications. A major challenge and opportunity will lie in leveraging Paneth cell biology for personalized therapeutic strategies aimed at restoring epithelial integrity and improving outcomes in IBD patients.

## Data Availability

No datasets were generated or analysed during the current study.
